# 

**Published:** 1911-03-04

**Authors:** 


Just Issued. Ninth Edition, Rewritten and Enlarged, 9s. net.

PHARMACY, MATERIA MEDICA & THERAPEUTICS.

Being a Commentary on the B.P. and its Drugs, with their Preparations, and on all the new Non-official Remedies in use, with Chapters on the different processes used in Dispensing, Prescription-writing, Latin Glossary, &c. By Sir W. WHITLA, M.D., LL.D., Professor, Materia Medica, Queen's University, Belfast, and Senior Physician, Royal Victoria Hospital.

By the same Author. New Work. 1,900 pages. Crown 8vo. 25s. net.

PRACTICE OF MEDICINE.

Containing in two Handy Volumes a complete and independent Article on the Etiology, Symptoms, and Diagnosis of every Medical Disease; arranged in Dictionary Form for Practitioners and Students.

London: Baillière, Tindall & Cox, 8 Henrietta Street, W.C.

### OBITUARY.

### PROFESSOR VAN CAUWENBERGHE.

As the result of an unfortunate accident Professor Charles Van Cauwenberghe died at Ghent on February 12. The deceased in mistaking a door in the municipal building of Olsene, whither he had been summoned in consultation, for the one leading to the telephone room, was precipitated down a stone staircase into the cellar. He recovered sufficiently to be able to return to his home, but rapidly grew worse after arrival there, and died the following morning in a state of coma. His career was one of exceptional success both professionally and socially. He had been rector of the University of Ghent, had held the post of vice-president, and was actually entering into his year of office as President of the Royal Academy of Medicine of Belgium when he met with the accident which caused his death. In 1908 he had been promoted to the rank of Commander of the Order of Leopold.

The death is announced of Dr. Charles Alexander MacMunn, of Wolverhampton, who was born in 1852 and educated at Trinity College, Dublin. He was honorary consulting physician to the Royal Orphanage, Wolverhampton, and devoted himself largely to research, mostly in physiological chemistry, and animal pigment. He was a Volunteer, and on the outbreak of war in South Africa in 1900 his services were accepted for hospital work.

### MEDICAL APPOINTMENTS, ETC.

L. E. Barrington Ward, M.B., F.R.C.S.Edin., has been appointed resident medical superintendent at the Hospital for Sick Children, Great Ormond Street, W.C.

Mr. L. S. B. Tasker, M.B., B.S., has been appointed house surgeon at the Evelina Hospital for Sick Children.

Dr. Ernest Hastings Tweedy has been elected a consulting gynæcologist to the Rotunda Hospital, Dublin.

### Phlyctenular Conjunctivitis.

This is a disease of ill-nourished children, most common in those between four and six years of age; it is much less frequent among Jewish children, who are better fed and get a great deal of oil with their food. We may here note that cod-liver oil is the best cure for the disease. As to the cause of the disease, I have put forward evidence to show that it is not the effect of a specific microbe, but that it is an efflorescence or herpetiform eruption on the terminals of the first and second divisions of the fifth cranial nerve, caused by peripheral irritation of collateral branches of the second division of that nerve by septic teeth, and nasal and labial sores in ill-fed children. This disease accounts for more cases of damaged eyes than any other, and no better argument for instruction in oral cleanliness and dental clinics can be made than the citation of this affection.—*Mr. N. Bishop Harman.*

### Gifts and Bequests.

The King has been graciously pleased to become a subscriber of £10 10s. to the Metropolitan Convalescent Institution.

Miss Mary Ashworth, of Rossmore, Boscombe, Hants, left £200 in her will to the Royal Infirmary, Manchester; £100 each to the Children's Hospital, Clifford Street, Manchester, the Hospital for Incurables, Manchester, St. Mary's Hospital, Manchester, Southport Infirmary, Rochdale Infirmary, the Burnley Infirmary, Bury Infirmary, Huddersfield Infirmary, the Victoria Hospital, Bournemouth, Bournemouth Sanatorium, and the Boscombe Hospital, Boscombe.

To commemorate her visit to the Gravesend Hospital on July 20 last, when she received purses in aid of the funds amounting to over £1,000, Her Royal Highness Princess Alexander of Teck has allowed the Committee to name one of the women's wards after her. The ward will henceforth be known as the "Princess Alice" ward.

The workmen of Swanscombe Works of Associated Portland Cement Manufacturers, Ltd., in the area served by the Gravesend Hospital have forwarded to the Secretary of that institution a cheque for £200 out of their weekly hospital collections.

At the next meeting of the St. Pancras Borough Council the following motion will be considered: "That this Council regrets the laxity of various hospitals in allowing cases of an infectious nature, after post-mortem examinations, to be sent to their homes instead of to the public mortuaries."

### Jottings.

Lord Rothschild has sent a gift of hares to the Royal Hospital for Incurables, Putney Heath.

The Countess of Dalhousie will open a three-day Sale of Work at the Royal Hospital for Incurables, Putney Heath, on Tuesday, June 13 at 2.30 p.m.

The Board of Management of the General Infirmary, Chester, have called in Mr. Paul Waterhouse, M.A., F.R.I.B.A., to give advice in consultation with their architect, Mr. W. T. Lockwood, in regard to the Infirmary buildings.

The Right Hon. Sir Frank Cavendish Lascelles, G.C.B., will preside at this year's Anniversary Festival of the German Hospital, Dalston, which has been fixed for Friday, May 12, at the Whitehall Rooms, Hôtel Métropole.

The Annual General Meeting of the Governors and subscribers of the West Ham and Eastern General Hospital, Stratford, E., was held in the Council Chamber, Town Hall, Stratford, on Wednesday, March 1, at 8 p.m., Thos. Alex. Cook, Esq., presiding.

A Radium Institute for radiation and emanation therapy has been established at Berlin in connection with the Sanatorium, Koniggrätzer Strasse 105. Among the staff are Professor Anton Sticker (malignant diseases), Dr. Pritzel (diseases of metabolism) and Dr. Dreuw (skin diseases).

The League of Mercy.—Tuesday, March 7: Dulwich-Norwood District—Mrs. Edmunds, at home, Antron, Upper Tulse Hill, 4 p.m. Wednesday, March 8: Hampstead District—Mrs. Metzler, at home, 83 Avenue Road, N.W., at 3.30 p.m. Thursday, March 9: South Paddington District—Lady Dimsdale, at home, 79 Lancaster Gate (by kind permission of Mrs. E. Mackintosh) at 3.30 p.m.

The annual general meeting of the Royal Dental Hospital, Leicester Square, London, will be held at the hospital on Thursday, March 9, 1911, at 5.30 p.m., Mr. E. Lloyd-Williams, a vice-president of the hospital, in the chair, to receive the annual report, and elect the committee of management, the treasurer, and auditors for the ensuing year.

Gratifying records of increased subscriptions were presented at the sixth annual meeting of the Bradford Hospital Fund (Incorporated) which was held last Thursday. The contributions from the workers in local mills and workshops again showed an increase, the amount collected from these sources being £4,724, as against £4,089 last year. The total amount received by the Fund was £7,328, and the amount received in the previous year was £6,963 11s. 6d.

A new organ for the services at the Royal Hospital for Incurables, Putney Heath, is needed, as the instrument now in use, after forty years' work, is defective and beyond repair. The estimated cost of an adequate instrument for the large assembly-room is £400, and as the Board of Management have no special fund, help is earnestly sought. The present organ was a second-hand one when it was presented to the Institution by a former treasurer.

By the kindness of Mr. Oswald Stoll the Leicester Palace Theatre has again been offered to the Leicester Infirmary and Children's Hospital for a matinée to be held on Thursday afternoon, April 20. A strong committee has been formed to organise the effort with the Deputy-Mayor (Councillor Chitham) president, Mr. Herman Wildt chairman, Mr. T. H. Prentice vice-chairman, Mr. Arthur Baum hon. treasurer, and Messrs. G. F. Reynolds and J. H. Ward hon. secretaries. As the result of last year's matinée £115 was handed over to the infirmary.

Sir Algernon Coote, Bart., and Dr. Ernest Hastings Tweedy, F.R.C.P.I., ex-master of the hospital, have been elected Governors of the Rotunda Hospital, Dublin.

### THE WEEK'S NOVELTIES.

THE **LEMCO and OXO COMPANY** have now selected the whole of their 1,200,000 acres of land in Rhodesia. Situated 3,000 feet above sea level, in a delightful climate abounding with luxuriant pasture, the Lemco and Oxo ranches in Rhodesia will enable the Company to continue their policy of open-air breeding. Experience has proved that this ensures practical immunity from tuberculosis, which is impossible where cattle have to be housed in sheds as in this country. It is the proud boast of the Lemco and Oxo Company that it is entirely independent of outside sources of supply for raw material. All beef used comes from the Company's own cattle, examined by expert veterinaries immediately before slaughter and immediately after. It is thus a practical impossibility for anything but healthy beef to be used in the Lemco and Oxo products. Including the Argentine and other farms the total acreage of the Lemco and Oxo farms now amounts to nearly 5,000,000 acres.

HUNTLEY AND PALMER'S **AKOLL BREAD** contains a remarkably low percentage of carbohydrates, and a large percentage of proteins and fats. It is very appetising and palatable, and, together with Akoll Biscuits, forms an important item in the up-to-date dietary of the diabetic.

THOS. CHRISTY AND CO., of Old Swan Lane, are the sole agents in this country for **GLYCO-THYMOLINE**, pleasant non-irritating anti-catarrh preparation for internal and external use. As an ingredient in simple mixtures for children this preparation has already won the favour of many practitioners. Those who are not yet acquainted with it should write for a sample, which is sent post free on application to the agents.

### ANSWERS TO CORRESPONDENTS.

A.S.W.—You do not say anything about your preliminary studies, whether you are a matriculated student of a university or not. If you are not, this is the first step —to matriculate or pass a preliminary examination in arts. It is useless to attempt to study specific parts of the curriculum before that. There is no good handbook on the subject, but you will obtain particulars of courses and advice generally by writing to the Dean of any London or Provincial medical school.

L.M.—We cannot recommend particular medical schools. You must choose for yourself after examining the special advantages of each school. A large school is not necessarily a good school.

ENQUIRER.—There are many adhesive plasters in the market. One of the best we know of is Leukoplast, which seems specially suited for the purpose you require. It is quite strong enough for extension in cases of fracture of the femur and we have used it for that purpose. Write to Messrs. Beiersdorf & Co., 7 Idol Lane, E.C., for sample and literature.

### BUILDINGS CONTEMPLATED AND IN PROGRESS.

GELLIGAER.—The new infectious hospital, which is estimated to cost £12,000, and for which Mr. D. Vivian Jones, of Hengoed, is architect, will be situated on elevated ground a little over a mile from Pengam. It will consist of two pavilion blocks, each containing male and female wards with five beds, and one containing two single-bed separation wards. There will also be an administration block, laundry, entrance lodge and stabling.

ILFORD.—Mr. W. A. Pite, F.R.I.B.A., is architect for the new mortuary block to Dr. Barnardo's Homes Hospital.

MACCLESFIELD.—Tenders have just been received for alterations to the Macclesfield Infirmary. Mr. N. Hartley Hacking, of Manchester, is the architect. The lowest tender is £450 by Messrs. Roylance & Co., Ltd., of Macclesfield.

### MEDICAL APPOINTMENTS.

FULL particulars of these vacancies can be obtained on reference to our advertisement columns :—

BIRMINGHAM AND MIDLAND HOMŒOPATHIC HOSPITAL.—Resident House Surgeon.

CUMBERLAND INFIRMARY, CARLISLE.—Resident Medical Officer.

HULL ROYAL INFIRMARY.—Assistant House Surgeon. Casualty House Surgeon.

TOWER HAMLETS DISPENSARY.—Resident Medical Officer.

WALSALL AND DISTRICT HOSPITAL.—Junior House Surgeon.

**THE SCIENTIFIC PRESS PUBLICATIONS**

**MEDICAL HISTORY FROM THE EARLIEST TIMES.**

By E. T. Withington, M.A., M.B. Oxon. Demy 8vo. over 400 pages, with 2 Plates, cloth gilt, 12s. 6d. net; 12s. 10d. post free.

**PHOTOMICROGRAPHY.**
By Edmund J. Spitta, L.R.C.P. Lond.; M.R.C.S. Eng., F.R.A.S. Demy 4to. with 41 Half-tone Reproductions from Original Negatives and 63 Illustrations in the Text, cloth gilt, 12s.

**THE SCHOTT TREATMENT FOR CHRONIC HEART DISEASES.**

By Richard Greene, F.R.C.P. Ed. Second Edition, Revised, demy 8vo. paper cover, 6d.

**THE MENOPAUSE AND ITS DISORDERS.**
With Chapters on Menstruation. By A. D. Leith Napier, M.D., M.R.C.P. Royal 8vo. cloth gilt, illustrated by a series of Original Photo-micrographs and many Illustrations in the Text, 7s. 6d. net; 7s. 11d. post free.

**THE CONSUMPTIVE WORKING MAN: What can Sanatoria do for him?**

By Noel Dean Bardswell, M.D., Medical Superintendent, King Edward VII. Sanatorium, Midhurst. Demy 8vo. illustrated, cloth, 10s. 6d. net; 10s. 10d. post free.

**SANATORIUM TREATMENT OF CONSUMPTION.**
By T. N. Kelynack, M.D., M.R.C.P. Crown 8vo. limp cloth cover, 6d. net; 7d. post free.

**THE SELECTION OF CONSUMPTIVE CASES for SANATORIUM TREATMENT.**
By T. N. Kelynack, M.D., M.R.C.P. Crown 8vo. limp cloth cover, 6d. net; 7d. post free.

**CLINICAL DIAGNOSIS:**
A Practical Handbook of Chemical and Microscopical Methods. By W. G. Aitchison Robertson, M.D., F.R.C.P. Edin. Fcap. 8vo. profusely Illustrated, cloth gilt, 3s. 6d. net; 3s. 9d. post free.

*OBTAINABLE OF ALL BOOKSELLERS, OR OF*

**THE SCIENTIFIC PRESS, LTD., Medical and Scientific Publishers,**

### GENERAL PRACTITIONERS' CONTRIBUTIONS.

The Hospital devotes a special page to General Practitioners' contributions. We therefore invite from practitioners contributions based upon their experience in the management of cases, and in the treatment and diagnosis of disease; especially shall we be prepared to welcome articles dealing, practically, with treatment, and with the use and value of new remedies and methods.

No article should exceed 1,100 words, and, if accepted, one guinea for a contribution of this length will be paid to the writer after publication. Each communication should be accompanied by a stamped directed envelope for the return of the MS. if found unsuitable.

Contributions should be written, or preferably typed, on one side of the paper only, and all articles sent in are accepted upon the distinct understanding that they are forwarded to The Hospital only.

### The Relaxations of Medical Men.

We shall also be glad to pay for accepted contributions, from any member of the profession, on the subject of the relaxations of practitioners. This opens up a wide field, as it includes natural history, photography, sport, indoor recreations, and motoring. Whenever possible, original illustrations and photographs should be sent with the MS.

### Suggestions Invited.

The Editor will welcome suggestions for the establishment of any new section in The Hospital, and will be glad to supply information on any subject of interest or importance to members of the profession in any part of the world.

### Books for Review.

Publishers are particularly requested to send advance proofs of any new books of importance, whenever possible, as the Editor has made arrangements to publish immediate reviews, and on a new plan.

### Correspondence.

Correspondence on all subjects is invited, but no communication can be entertained if the name and address of the correspondent are not given as a guarantee of good faith, but not necessarily for publication. All correspondents should write on one side of the paper only.

### BUSINESS NOTICES.

### Annual Subscriptions.

The rate of annual subscriptions to The Hospital, either direct from the Office or through agents, is:—

For the United Kingdom ... ... 15s. a year
For the Colonies and Abroad ... 17s. ,,
For the United Kingdom (with Insurance Policy) ... ... ... £1 ,, inclusive of postage in each case.

Letters relating to the Publishing, Sale and Advertisement Departments must be addressed to the Manager (*not to the Editor*):—

The Manager,
The Hospital Building,
28 and 29 Southampton Street,
Strand, London, W.C.

